# Turner Syndrome with Mosaicism of X Chromosome: A Case Report

**DOI:** 10.31729/jnma.8168

**Published:** 2023-05-31

**Authors:** Priyanka Vaidya, Achala Vaidya

**Affiliations:** 1 Department of Obstetrics and Gynaecology, Toowoomba Hospital, Toowoomba City QLD 4350, Queensland, Australia; 2 Department of Obstetrics and Gynaecology, Norvic International Hospital, Thapathali, Kathmandu, Nepal

**Keywords:** *case reports*, *infertility*, *sex chromosome aberrations*, *Turner syndrome*

## Abstract

Turners' syndrome, although common, is a complex syndrome that requires a multidisciplinary team to manage it. If undiagnosed prenatally or in childhood, Turners' syndrome females often present later to gynaecologists with premature ovarian insufficiency or infertility as their primary presenting complaint. Timely diagnosis and management are key to improving health outcomes in women with Turners' syndrome, as it is associated with multiple comorbidities which left untreated will result in excess morbidity and mortality. We hereby present a case of a 20-year-old female diagnosed to have Turner's syndrome with mosaicism of the X chromosome to highlight the wide array of clinical presentations it can have.

## INTRODUCTION

Turner Syndrome is a sex chromosome abnormality in females, affecting 1/2000 to 1/2500 live female births.^1^ It is associated with partial or complete loss of the second X chromosome in phenotypic females, with the loss of the paternally derived X chromosome in 75-80% of cases.^2^ The absence or mosaicism of the X chromosome results in gonadal dysgenesis. We present a case of a 20-year-old female with turner's syndrome (TS) with mosaicism of the X chromosome to highlight the wide array of clinical presentations it can have. Typical features include short stature and premature ovarian failure in a phenotypic female. It represents an important cause of ovarian insufficiency and estrogen deficiency secondary to hypogonadism in females. It is critical a timely diagnosis is made, so that treatment can be commenced to optimise growth, maintain pubertal development, and prevent comorbidities such as severe osteoporosis.

## CASE REPORT

We present the case of a 20-year-old female who presented with secondary amenorrhea for 8 months. She commenced her menarche at age 12 years and had regular cycles for 3 years, which then consequently became irregular. She had normal flow for 4-5 days, without menorrhagia or dysmenorrhea. She had no past medical or surgical history and no family history of significance.

On examination, she had some characteristic turner features including short stature, wide carrying angle and widely spaced nipples. Her abdominal examination and external genitalia examination were unremarkable.

On investigation, blood tests revealed an elevated follicle-stimulating hormone (FSH) of 41.2 mmol/ml, low estradiol of 14.12, low progesterone of <0.10 and low testosterone of 0.07 mmol/ml. The remaining hormone profile was unremarkable with a prolactin of 170 and DHEAS of 5.5 mmol/L. An ultrasound of the abdomen pelvis revealed normal anatomy with streak ovaries.

Chromosome analysis revealed a normal X chromosome and an isochromosome of the long arm of the second X chromosome at band Xq10. The abnormal chromosome complement was consistent with the diagnosis of variant Turner Syndrome.

After the diagnosis was made, she was prescribed three cycles of oral contraceptive pills. Follow-up was done two times. On further follow up, she needs to be reassessed for ability to conceive and proper counseling should be done.

## DISCUSSION

The presentation of Turner syndrome varies throughout the individual's life. During the antenatal period, the diagnosis should be considered in a female fetus with hydrops, increased nuchal translucency, cystic hygroma or lymphedema.^3^ Neonates have congenital lymphedema, webbed neck, nail dysplasia, narrow high arched palate and short fourth metacarpals/metatarsals.^4^ For adolescents and adults, the key clinical features include lack of secondary sexual characteristics including breast development, primary or secondary amenorrhea and infertility. In this case, the diagnosis was made in an adult female with typical features including short stature, widely spaced nipples (shield chest) and cubitus valgus.^5^

The diagnosis of TS is confirmed with standard karyotyping, which was performed in this case. More than half the patients have a missing X chromosome (45,X) or a combination of monosomy X and normal cells 45XO/46XX or 45XO/46XY mosaic. Additionally, other sex chromosome anomalies such as isochromosome Xq, ring X, deletion X or abnormal Y chromosome can also cause the condition.^5,6^

The 45X karyotype, the commonest type, causes TS females to have a more severe phenotype compared to women with mosaicism.^7,8,9^ Thus, diagnosis is often made prenatally or during childhood for 45X karyotype females, but delayed, for TS females with mosaicism. Delay in diagnosis may prevent the timely use of growth hormone for short stature or hormone replacement therapy (HRT) and identification of comorbidities that can negatively impact TS females.^10^

TS is associated with primary hypogonadism with accelerated loss of oocytes resulting in primary amenorrhea, pubertal arrest or premature ovarian insufficiency (POI). FSH and Anti-mullerian hormone (AMH) helps estimate ovarian function and predicts the need for estradiol replacement. HRT-containing estrogen is recommended for all TS females for either pubertal induction and/or management of POI till the expected age of menopause-approximately 51 years of age.

Low-dose estradiol therapy, preferred as transdermal preparation, is recommended to allow the normal pace of puberty and attain peak bone mass. A progestogen is advised two years post estradiol monotherapy or after the first vaginal bleed to optimize breast development and allow endometrial protection.^1,11^ Androgen levels are decreased in women with TS, as demonstrated in the case, which, negatively impacts their sexual function, neurocognition, and quality of life. Some studies claim the addition of methyl-testosterone in addition to HRT is beneficial.^1^

Infertility is common in most TS females; thus, pregnancy through assisted reproductive technology (ART) is often utilized. Spontaneous conception of pregnancy often witnessed in mosaic karyotypes, is also associated with an increased risk of miscarriage, maternal cardiovascular complications as well as neonatal risks including chromosomal or congenital abnormalities. Oocyte preservation is recommended for girls over 12 years due to the risk of POI.^1^

TS is associated with multiple comorbidities associated with cardiovascular disease (aortic aneurysm, coarctation of the aorta and valvular disease), multiple autoimmune diseases (diabetes, thyroid disease, Crohn's/ulcerative colitis), skeletal anomalies (kyphosis, scoliosis, dysplastic hip) and renal anomalies. Studies show that the isochromosome Xq karyotype, as described in the case, has a higher association with diabetes mellitus. These co-morbidities have term consequences and require regular surveillance in adulthood.^1,12^
